# Rapid bioassay to measure early reactive oxygen species production in *Arabidopsis* leave tissue in response to living *Pseudomonas syringae*

**DOI:** 10.1186/1746-4811-10-6

**Published:** 2014-02-26

**Authors:** John M Smith, Antje Heese

**Affiliations:** 1 Division of Biochemistry, University of Missouri-Columbia, 117 Schweitzer Hall, Columbia, MO 65211, USA; 2 Division of Plant Sciences, University of Missouri-Columbia, Columbia, MO 65211, USA; 3 Interdisciplinary Plant Group (IPG), University of Missouri-Columbia, Columbia, MO 65211, USA

**Keywords:** ROS, Reactive oxygen, PAMP, FLS2, RbohD, BAK1, flg22, *Pseudomonas syringae*, DC3000, *hrcC*

## Abstract

**Background:**

*Arabidopsis thaliana* and *Pseudomonas syringae* pathovar *tomato* (*Pto*) provide an excellent plant-bacteria model system to study innate immunity. During pattern-triggered immunity (PTI), cognate host receptors perceive pathogen-associated molecular patterns (PAMPs) as non-self molecules. *Pto* harbors many PAMPs; thus for experimental ease, many studies utilize single synthesized PAMPs such as flg22, a short protein peptide derived from *Pseudomonas* flagellin. Flg22 recognition by *Arabidopsis* Flagellin Sensing 2 (FLS2) initiates a plethora of signaling responses including rapid production of apoplastic reactive oxygen species (ROS). Assessing flg22-ROS has been instrumental in identifying novel PAMP-signaling components; but comparably little is known whether in *Arabidopsis*, ROS is produced in response to intact live *Pto* and whether this response can be used to dissect genetic requirements of the plant host and live bacterial pathogens *in planta*.

**Results:**

Here, we report of a fast and robust bioassay to quantitatively assess early ROS in *Arabidopsis* leaves, a tissue commonly used for pathogen infection assays, in response to living bacterial *Pto* strains. We establish that live *Pto* elicits a transient and dose-dependent ROS that differed in timing of initiation, amplitude and duration compared to flg22-induced ROS. Our control experiments confirmed that the detected ROS was dependent on the presence of the bacterial cells. Utilizing *Arabidopsis* mutants previously shown to be defective in flg22-induced ROS, we demonstrate that ROS elicited by live *Pto* was fully or in part dependent on *RbohD* and *BAK1*, respectively. Because *fls2* mutants did not produce any ROS, flagellin perception by FLS2 is the predominant recognition event in live *Pto*-elicited ROS in *Arabidopsis* leaves. Furthermore using different *Pto* strains, our *in planta* results indicate that early ROS production appeared to be independent of the Type III Secretion System.

**Conclusions:**

We provide evidence and necessary control experiments demonstrating that *in planta*, this ROS bioassay can be utilized to rapidly screen different *Arabidopsis* mutant lines and ecotypes in combination with different bacterial strains to investigate the genetic requirements of a plant host and its pathogen. For future experiments, this robust bioassay can be easily extended beyond *Arabidopsis*-*Pto* to diverse plant-pathosystems including crop species and their respective microbial pathogens.

## Background

Eukaryotes have developed highly effective immune mechanisms for protection against microbial pathogens using pattern-triggered immunity (PTI) as the first line of defense. Pathogen-associated molecular patterns (PAMPs), also referred to as microbe-associated molecular pattern (MAMPs), are highly conserved and essential molecules common to entire classes of microbes but are absent from the host. Host cells utilize pattern recognition receptors (PRRs) to recognize PAMPs as non-self to initiate a large number of signaling responses that contribute to growth restriction of microbial pathogens [1-3]. To evade these host immune responses, pathogenic microbes express and deliver effector molecules into host cells to interfere with PTI [4]. For example, the virulent model bacterium *Pseudomonas syringae* pathovar *tomato* (*Pto*) DC3000 translocates 28 or more effector proteins into plant cells via the type III secretion system (T3SS), some of which are known to suppress PTI [5-7]. Some effector proteins, however, betray the pathogen due to their direct or indirect recognition by cytosolic host resistant proteins resulting in Effector-Triggered Immunity (ETI) [1,5-8]. Non-pathogenic strains lacking functional T3SS such as *Pto* DC3000 *hrcC*^*-*^ (*Pto hrcC*^*-*^) do not suppress PTI because of their inability to deliver effectors into host cells [6,8].

In plants, only few PAMP/PRR pairs involved in PTI have been identified and characterized [1,2,9,10]. In the model plant *Arabidopsis thaliana*, the best studied PTI-system is perception of bacterial flagellin by Flagellin Sensing 2 (FLS2), a plasma membrane localized PRR [2,11]. Flagellin is the main proteinaceous component of extracellular flagellum filaments essential for the mobility and ability of bacteria such as *Pto* to infect host plants [12,13]. Binding of flagellin or flg22, a 22-amino acid peptide derived from the consensus sequence for the most highly conserved region in the N-terminus of eubacterial flagellin [14], to the extracellular domain of FLS2 occurs within the plant apoplast and leads to a variety of early and late signaling responses [11,15,16], all of which are dependent on BRI1-Associated Receptor Kinase 1 (BAK1) [17,18]. One of the best characterized and robust early PAMP-signaling events is the rapid and transient accumulation of apoplastic reactive oxygen species (ROS). Assessing PAMP-elicited ROS has proven to be a valuable tool in identifying and characterizing novel PAMP-signaling components and specific amino acids necessary for their function [18-23]. Production of rapid apoplastic ROS in response to PAMP peptides is solely dependent on the plasma membrane localized NADPH respiratory burst oxidase homolog D (RbohD) [22,24,25]. Although the exact role of ROS production in innate immunity remains unclear, ROS initiates a plethora of downstream signaling events, some of which are essential in establishing defense mechanisms to prevent the spread of bacterial pathogens [26,27].

Over the past decade, studies utilizing commercially synthesized flg22 peptide have greatly aided in increasing our insight into early and late signaling events and in identifying required signaling components and their contribution to PTI [2,28]. Other efforts exploited boiled bacterial extract to investigate various PAMP-induced responses in leaves or cultured plant cells [14,29-31]. Boiling bacterial cells results in the release of both extra- and intracellular PAMPs, thus making it difficult to determine the biologically relevant order of PAMP recognition by specific host PRRs. The disadvantage of utilizing cultured cells as opposed to leaf tissue is that because of the unavailability of *Arabidopsis* mutant cell culture lines, cell culture limits the ability to assess the genetic requirement of the plant host for the response of interest. In contrast, the genetic plant host-pathogen interplay between *Arabidopsis* plants and *Pseudomonas* can be interrogated *in planta* due to the availability of large number of *Arabidopsis* ecotypes and mutant lines. When investigating responses induced by living bacteria in plant tissue (*in planta*), most efforts have focused on later responses such as accumulation of the defense hormone salicylic acid (SA) [12-24 hours post infection (hpi)], transcriptional changes of the late gene marker *PR1* (24 hpi) or changes in resistance to bacterial infection measured 3 days post-infection (dpi). Only more recently, attention has been given to identifying early signaling events and their genetic requirements induced by living bacterial pathogens on plant host leaves [32-35], the tissue that serves as the primary source for bacterial pathogen infection.

Here, we describe advancement of a fast and convenient *in planta* bioassay that allows for quantitative assessment of early ROS production in *Arabidopsis* leaf tissue, the primary site of *Pto* infection, induced by living *Pto* strains. Importantly, we provide necessary control experiments showing that *in planta*, early ROS production was dependent on the presence of *Pto* cells. By utilizing *Arabidopsis* mutants previously shown to be affected in flg22-induced ROS production, we demonstrate that early ROS produced in response to live *Pto* strains was fully dependent on *RbohD* and *FLS2* and partially dependent on *BAK1*. No statistical differences were observed between ROS induced by *Pto* DC3000 and *hrcC*^-^ cells, thus the virulence-promoting T3SS does not appear to have an influence on early ROS production. Because of the ease in setting up ROS assays in a 96-well plate assay, this quantitative analysis is highly suitable to screen within a relatively short period of time large populations of *Arabidopsis* accession lines in combination with diverse *Pto* mutant strains to define the genetic requirements of host and bacterial pathogen.

## Results and discussion

**Figure 1:**
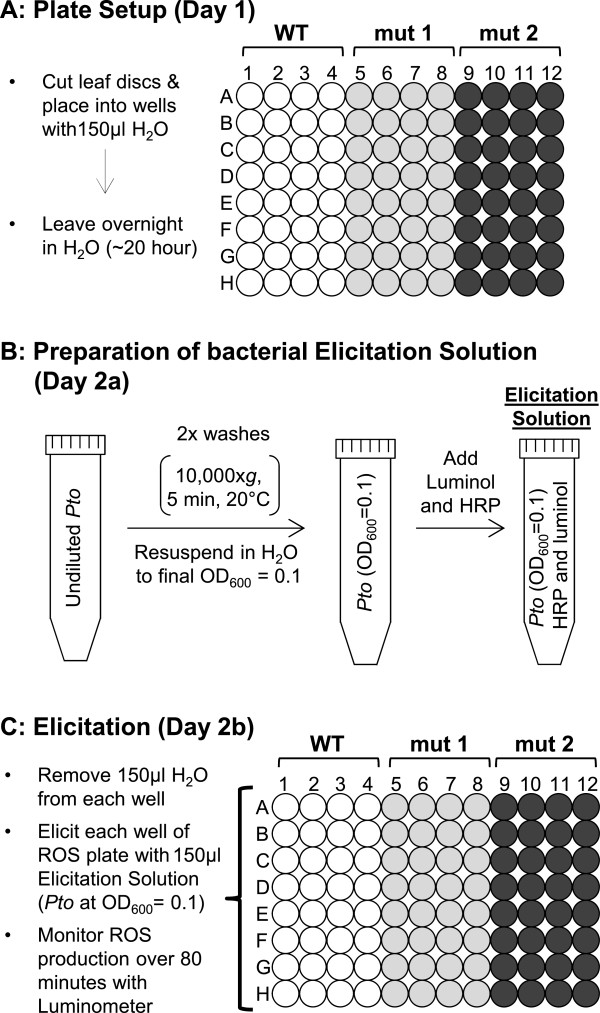
**Outline for plate-setup and preparation of bacterial elicitation solution to measure luminol-based ROS production in leaf disc halves of *Arabidopsis *wildtype (Col-0) and mutant lines in response to live bacterial *Pto *cells. A**: Preparation of a 96-well assay plate containing leaf disc halves of wildtype (WT, white) and mutant *Arabidopsis* lines (mut 1, gray and mut 2, black). **B**: Preparation of Elicitation Solution containing live *Pto* cells. **C**: Elicitation of wild-type and mutant tissue with Elicitation Solution to measure ROS response. WT, wild-type; mut, mutant; OD, optical density.

Our goal was to establish a rapid and robust *in planta* method to quantitatively measure early ROS production in response to live *Pto* strains in *Arabidopsis* leaves, a tissue commonly used for bacterial infection assays. To this end, we adapted the luminol-based ROS assay used for PAMP-elicitation [18,21,36,37] in that we substituted the synthetic PAMP peptide with bacterial cells of *Pto* strains that were freshly grown for 36-48 hours at 22°C on King’s B media (KBM) plates containing appropriate antibiotics [Figure 1; see Methods for details]. In brief, *Arabidopsis* leaf discs obtained from 5 week old plants were cut in half with a sharp razor blade to increase surface area, and each half was placed into a distilled water (dH_2_O)-containing well of a 96-well titer plate for at least 20 hours to reduce wounding effect [Figure 1A]. Immediately prior to elicitation, bacteria were harvested directly from plates [38] and washed twice in sterile dH_2_O. After the second wash step, bacterial cells were resuspended in sterile dH_2_O [38], and their cell densities (OD_600_) were adjusted between OD_600_ = 0.001 to OD_600_ = 0.1 [Figure 1B]. The OD_600_ can be used as a rapid means to provide an estimation of bacterial cell density (as colony-forming units (cfu)/mL) [38,39]. After Luminol and Horseradish Peroxidase (HRP) was given to the *Pto*-containing solution, this Elicitation Solution was added to *Arabidopsis* leaf halves to measure luminol-based ROS production between 0 and 80 minutes using a luminometer [Figure 1C].

**Figure 2:**
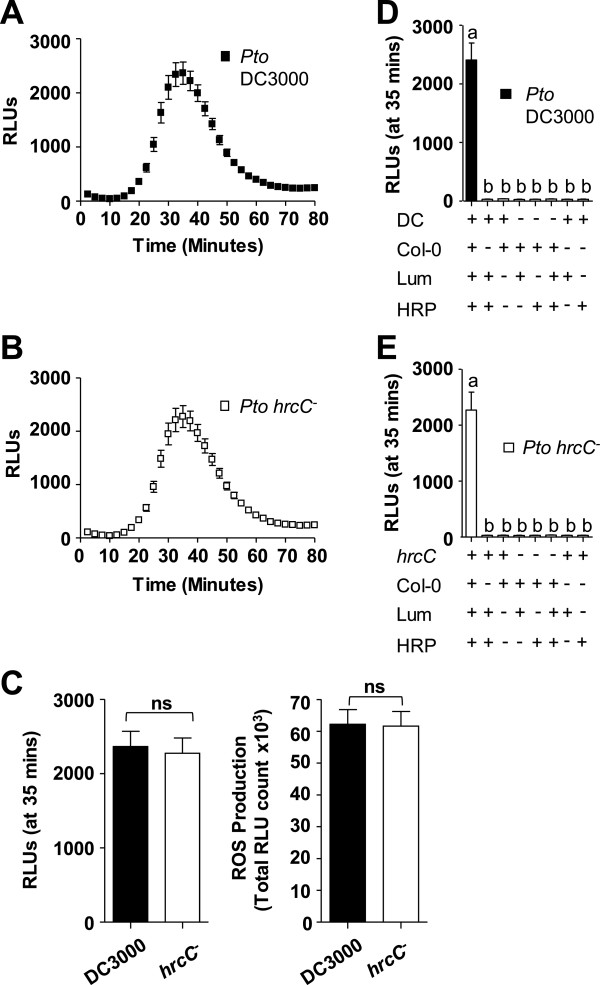
**Transient ROS production in response to live *Pto *DC3000 and *Pto hrcC***^***-***^**in wildtype *Arabidopsis *Col-0. A**: Time-course of ROS production in response to *Pto* DC3000 (n = 48/treatment). **B**: Time-course of ROS production in response to *Pto hrcC*^*-*^ (n = 48/treatment). **C**: ROS production induced by *Pto* DC3000 (black bar) and *Pto hrcC*^*—*^(white bar) shown at peak ROS production (35 minutes post-elicitation) and as total Relative Light Units (RLUs) over 80 minutes following elicitation with indicated bacteria (n = 48/treatment). **D**: Specificity of ROS production based on presence of *Arabidopsis* tissue and *Pto* DC3000 cells (n = 12/treatment). **E**: Specificity of ROS production based on presence of *Arabidopsis* tissue and *Pto hrcC*^*-*^ cells (n = 12/treatment). To allow direct comparisons, ROS experiments in **(A, B and C)**, in **D** or in **E** were performed in the same 96-well plate at the same time. Values are mean ± SE, means with different letters denote a significance difference while similar letters denote no significance (Two tailed student’s *t*-test, P < 0.0001). For all experiments, bacterial solutions were used at OD_600_ = 0.1. Experiments were repeated more than 3 times with similar results. RLU, Relative Light Units; ns, no significance; DC, *Pto* DC3000; hrcC, *Pto hrcC*^*-*^; Lum, Luminol; HRP, Horseradish Peroxidase.

First, we established that virulent live *Pto* DC3000 cells induced ROS-production in wild-type *Arabidopsis* Col-0 leaves. As shown in Figure 2A, ROS production after elicitation with *Pto* DC3000 (OD_600_ = 0.1) was transient, in that increases in ROS was first detected around 20 minutes, peaking around 35-40 minutes and returning to near-basal levels around 70 minutes post-elicitation. Thus, it was delayed compared to that of PAMP peptide flg22-induced ROS which can be detected within 2-4 minutes, peaks at 10-14 minutes and returns to basal levels around 30-35 minutes [Additional file 1]. An explanation for the delayed ROS response to *Pto* DC3000 cells may be that in contrast to pure and short PAMP peptide(s), the PAMP(s) derived from living bacterial cells may not be readily accessible but may need to be released and/or processed for perception by the plant PRR(s) as suggested for flagellin-FLS2 system [40]. The reduced amplitude in response to *Pto* DC3000 cells may be an indication that living bacteria possibly release lower concentrations of PAMP(s) into the host apoplast compared to the concentration of applied synthesized flg22 peptide.

It is also possible that the reduced *Pto* DC3000-induced ROS amplitude may be due to suppression of ROS by bacterial effectors delivered into host cells. To address this hypothesis, we measured ROS production in response to non-pathogenic *Pto hrcC*^*-*^ cells (OD_600_ = 0.1) that lacked functional T3SS and are defective in effector delivery [Figure 2B]. Importantly, ROS induced by *Pto hrcC*^*-*^ was similar in the time of initiation, amplitude and duration compared to that by virulent *Pto DC3000* [Figure 2B; also see Figure 2C]. ROS production at their peaks (at 35 minutes) and total ROS productions over the 80 minute time-course did not show any statistically significant difference between these two *Pto* strains [Figure 2C], indicating that this *Pto*-elicited ROS production is independent of a functional T3SS. These results also suggest that compared to PAMP-induced ROS, the lower level ROS amplitude in response to *Pto* cells is unlikely due to interference of ROS by bacterial effectors. Similar ROS results were obtained over 75 min time-course after treatment with *Pto avrRpm1* and *Pto avrRps4*, two avirulent bacterial strains known to inject the avirulence proteins AvrRpm1 and AvrRps4, respectively, into host cells resulting in ETI-dependent responses [5-8]. Comparing *Pto avrRpm1* and *Pto avrRps4* to *Pto* DC3000, no difference in ROS initiation, amplitude and attenuation as well as total ROS production was observed over the 80 min time-course [Additional file 2]. Taken together, our results using virulent, non-pathogenic and avirulent *Pto* strains are in agreement that the observed ROS response was due to PTI-dependent events and are consistent with *Pto* effector delivery into host cells occurring at significantly later times post-infection (> 3 hours) [41] than the ROS response investigated in our study.

In control experiments, ROS production was measured using Elicitation Solutions that contained or lacked *Pto* strain (*Pto*), luminol, HRP and Col-0 leaf discs (Col-0) in different combinations. As evident in Figure 2D and E, ROS was produced only when the *Pto* strain, luminol, HRP and the leaf discs were present. Lack of any of these components did not result in any significant ROS production. No difference in the ROS production was observed in *Pto* strains resuspended in dH_2_O or 10 mM MgCl_2_ [Additional file 3].

**Figure 3:**
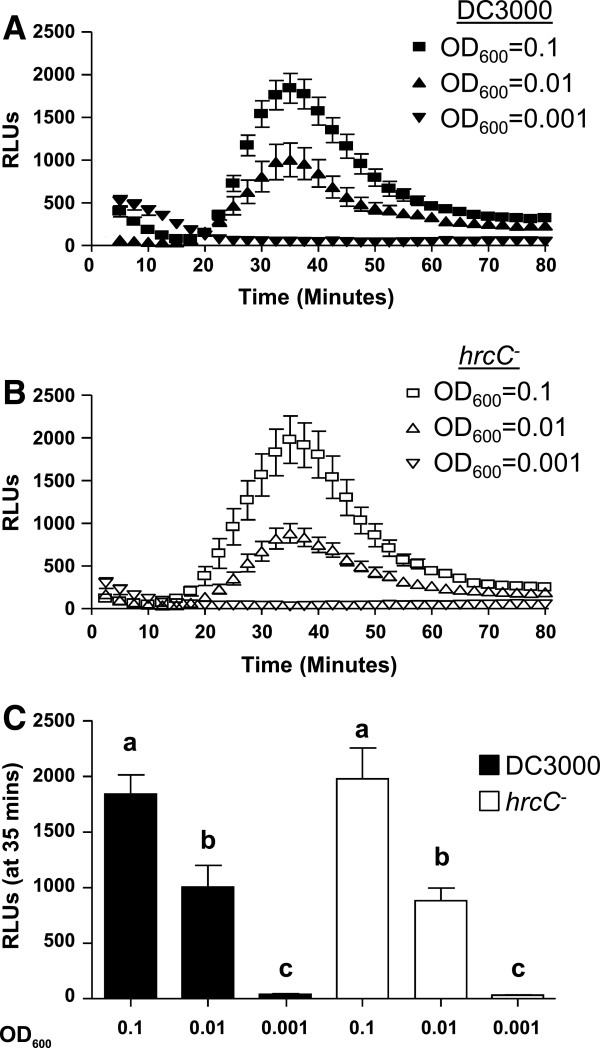
**Dose-dependent ROS production in response to live *Pto *DC3000 and *Pto hrcC***^***-***^**in wildtype Col-0. A**: Time-course of ROS production in response to *Pto* DC3000 cells with an OD_600_ of 0.001, 0.01 and 0.1. **B**: Time-course of ROS production in response to *Pto hrcC*^*-*^ cell with an OD_600_ of 0.001, 0.01 and 0.1. **C**: Comparison of peak ROS production for each concentration of *Pto* DC3000 (black bar) or *Pto hrcC*^*-*^ (white bar) shown at the point of maximum ROS production 35 minutes post-elicitation). For ROS production, Col-0 leaf disk halves were elicited with DC3000 (filled shape) or *hrcC*^*-*^ (open shapes) with the indicated bacterial cell density at 0 min (n = 16/treatment). To allow direct comparisons, all ROS experiments **(A-C)** were performed in the same 96-well plate at the same time. Values are mean ± SE, means with different letters denote a significance difference while similar letters denote no significance (Two tailed student’s *t*-test, P < 0.005). Experiments were repeated more than 3 times with similar results. RLU, Relative Light Units; OD, Optical density.

Next, we showed that *Pto*-dependent ROS production was dose-dependent by eliciting Col-0 leaf tissue with different bacterial cell densities between OD_600_ = 0.001 and 0.1 [Figure 3]. Either strain, *Pto* DC3000 or *Pto hrcC*^-^, elicited a ROS response in a dose-dependent manner [Figure 3A or B, respectively]. Consistent with Figure 2C, we did not observe any statistically significant difference when comparing ROS production elicited by *Pto* DC3000 and *Pto hrcC*^-^ at comparable optical densities [Figure 3C]. Furthermore, *Pto* optical densities of OD = 0.001 or below (data not shown) did not elicit any significant detectable ROS. *Pto* cells with an OD_600_ = 0.1 yielded the most consistent and robust ROS response, thus we used this bacterial cell density for all subsequent experiments.

**Figure 4:**
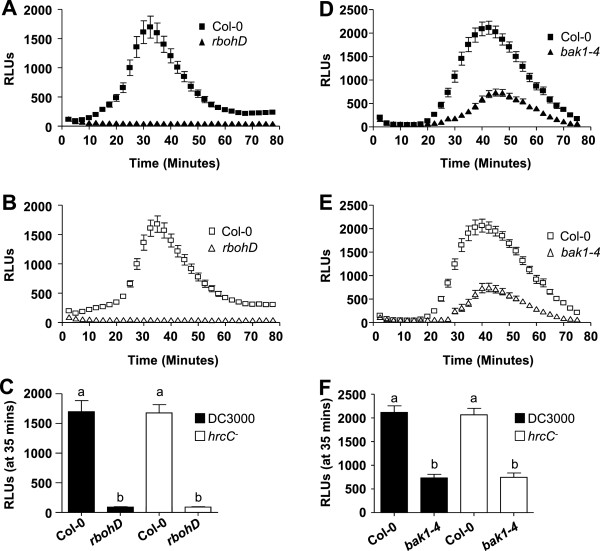
***RbohD *and *BAK1*-dependency of ROS production in response to live *Pto *DC3000 and *Pto hrcC***^***-***^**cells. A and B**: Time-course of ROS production in response to *Pto* DC3000 (**A**; closed symbols) or *Pto hrcC*^*-*^ (**B**; open sympols) in Col-0 (square) and *rbohD* null mutant (triangle) leaf disc halves (n = 24/treatment). **C**: Comparison of peak ROS production between *Pto* DC3000 or *Pto hrcC*^*-*^ in Col-0 and *rbohD* shown at the point of maximum ROS production (35 minutes post-elicitation from **A and B**). **D and E**: Time-course of ROS production in response to *Pto* DC3000 (**D**; closed symbols) or *Pto hrcC*^*-*^ (**E**; open sympols) in Col-0 (square) and *bak1-4* null mutant (triangle) leaf disc halves (n = 24/treatment). **F**: Comparison of peak ROS production between *Pto* DC3000 or *Pto hrcC*^*-*^ in Col-0 and *bak1-4* shown at the point of maximum ROS production (35 minutes post-elicitation from **D and E**). To allow for direct comparisons, all ROS experiments shown in **(A, B and C)** and **(D, E and F)** were performed in the same 96-well plate at the same time. Values are mean ± SE, means with different letters denote a significance difference while similar letters denote no significance (Two tailed student’s *t*-test, P < 0.0001). For all experiments, bacterial solutions were used at OD_600_ = 0.1. Experiments were repeated more than 3 times with similar results. RLU, Relative Light Units.

To investigate the genetic requirement of the plant host for *Pto*-induced ROS, we made use of previously characterized *Arabidopsis* mutants functioning in or upstream of apoplastic ROS production. A *rbohD* null mutant plant line in Col-0 background [22,25] was used to investigate dependency on *RbohD*, the plasma membrane localized protein previously shown to be the sole NADPH oxidase required for rapid induction of apoplastic ROS after elicitation with PAMP peptides [22,24,26,33]. In response to either live *Pto* DC3000 or *hrcC*^-^ cells, no ROS production was detected in *rbohD* compared to wild-type Col-0 for the 80 min time-course [Figure 4A or B, respectively; see also Figure 4C for peak comparison at 35 min post-elicitation]. We conclude that similar to the synthetic flg22 peptide, *Pto* DC3000 or *hrcC*^-^ elicited ROS production was fully dependent on *RbohD*. These result also suggested that the ROS measured in our assays was unlikely due to cell death-associated ROS.

Next, we determined whether *Pto*-elicited ROS was dependent on *BAK1*, the receptor-like kinase known to be required very early in initiating signaling after flg22-elicitation [18,20,42]. Underlining its crucial role in PAMP-signaling and PTI, BAK1 forms a ligand-induced receptor complex with FLS2 within seconds [18,20,42,43]. In response to *Pto* DC3000 or *hrcC*^-^, we observed an increase in ROS production over time in *bak1-4* null mutant leaf discs; but importantly, the ROS amplitude was significantly reduced compared to Col-0 [Figure 4D or E, respectively]. No statistical significant difference in ROS production was observed in *bak1-4* mutant tissue in response to *Pto* DC3000 or *Pto hrcC*^-^ [Figure 4F]. Taken together, these results suggest that *Pto*-elicited ROS was only in part dependent on *BAK1*. Our studies are consistent with previous reports showing that full signaling responses to the bacterial PAMP peptides flg22 require other proteins in addition to BAK1 [18,20,42].

**Figure 5:**
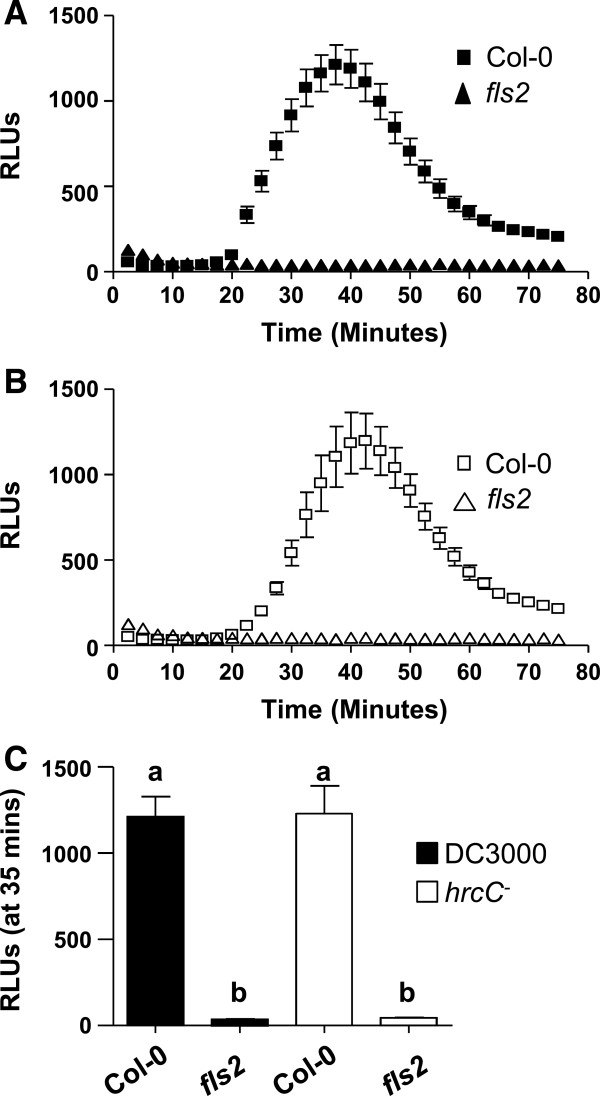
***FLS2*-dependency of ROS production in response to live *Pto *DC3000 and *Pto hrcC***^***-***^**cells.** Time-course of ROS production in response to *Pto* DC3000 (**A**; closed symbols) or *Pto hrcC*^*-*^ (**B**; open sympols) in Col-0 (square) and *fls2* null mutant (triangle) leaf disc halves (n = 24/treatment). **C**: Comparison of peak ROS production between *Pto* DC3000 or *Pto hrcC*^*-*^ in Col-0 and *fls2* shown at the point of maximum ROS production (35 minutes post-elicitation from **A and B**). To allow for direct comparisons, all ROS experiments shown in **(A, B and C)** were performed in the same 96-well plate at the same time. Values are mean ± SE, means with different letters denote a significance difference while similar letters denote no significance (Two tailed student’s *t*-test, P < 0.0001). For all experiments, bacterial solutions were used at OD_600_ = 0.1. Experiments were repeated more than 3 times with similar results. RLU, Relative Light Units.

Previous studies have shown that *Arabidopsis* mutants lacking functional FLS2 receptor are unable to perceive flg22 which results in a complete loss of any flg22/FLS2-dependent signaling event including lack of apoplastic ROS production [16,31,36]. In addition, FLS2-dependent responses contribute to restriction of *Pto* DC3000 growth [31]. However when treated with crude boiled *Pto* DC3000 extracts, *fls2* null mutants are still able to elicit signaling responses including ROS production [29,31]. These results are consistent with the idea that in addition to flagellin (flg22), *Pto* DC3000 contains multiple extra- and intracellular PAMPs that are exposed upon boiling of bacterial strains. To determine whether FLS2 contributed to ROS production induced by intact living *Pto* DC3000 or *hrcC*^-^, we utilized previously characterized *fls2* null mutants [18,31,36]. Interestingly, *fls2* null mutants in a Col-0 background did not elicit any ROS in response to either *Pto* strain [Figure 5A-C]. To confirm this observation, we challenged Wassilewskija (Ws-0), an *Arabidopsis* ecotype that is flagellin-insensitive and considered a natural *fls2* mutant due to an early stop mutation in the *FLS2* gene [31], with *Pto* DC3000 or *Pto hrcC*^-^. Similar to the *fls2* null mutant in Col-0, Ws-0 displayed a complete lack of ROS production in response to either *Pto* strains [Additional file 4]. Based on our studies utilizing two different *Arabidopsis* ecotypes lacking functional FLS2, the flagellin/flg22 receptor FLS2 appeared to be the predominant host receptor responsible for initiating early ROS production in response to intact, living *Pto* DC3000 and *hrcC*^-^. Our results are consistent with observations that in response to intact *Pto* DC3000 and DC3118 at 1 to 2 hours post-treatment, stomatal closure of epidermal leaf peels is entirely dependent on the presence of functional FLS2 [33,34]. Thus, these and our present studies suggest that not all potential *Pto* PAMPs appear to be exposed simultaneously during the initial bacterial infection. Our results further underline the advantages of utilizing living intact *Pto* cells in contrast to crude boiled bacterial extract [14,29,31] when studying early signaling responses induced by bacteria *in planta*. Boiling of bacteria likely releases both extra- and intracellular localized PAMPs simultaneously. In contrast, utilizing intact live bacterial cells enables researchers to determine the biologically relevant contribution and/or order of PAMP recognition by specific host PRRs.

## Conclusions

In this study, we report the advancement of a rapid and convenient bioassay allowing the quantitative assessment of ROS production between *Arabidopsis* leaf tissue (the primary site of *Pto* infection) and living *Pto* bacterial strains. Because of the ease in setting up ROS assays in a 96-well plate assay, this quantitative analysis is highly suitable to screen large populations of *Arabidopsis* accession lines in combination with diverse *Pto* mutant strains to define the genetic requirements of host and bacterial pathogen. In future experiments, this ROS bioassay may also allow addressing which PAMP/PRR pair quantitatively contributes to early signaling in response to bacterial pathogens in leave tissue. Furthermore, the utility of this bioassay can be easily extended beyond *Arabidopsis* and *Pto* to diverse model or crop species and their cognate microbial pathogen to define components required for early ROS responses.

## Methods

### Plant material and growth conditions

Arabidopsis seeds were sterilized with 10% bleach + 1% Triton X-100 for 1 hour, rinsed with water and plated aseptically on 0.5% agar containing 2.14 g L^-1^ Murashige and Skoog (MS) salts (Sigma Chemical Company, St. Louis, MO, USA, http://www.sigmaaldrich.com/) + 1% sucrose, pH 5.7. Following stratification for 2 days at 4°C, seedlings were germinated in Percival CU-36 L4 growth chambers (Percival, Perry, IA) under continuous light at 22°C [18]. Seven day-old seedlings were transplanted in soil and grown in an 8-h light/16-h dark photoperiod at 82 μmol m^-2^ s^-1^. Fully expanded rosette leaves were used from 4-5 week old plants for all ROS experiments. The Ws-0, *fls2Δ* (Col-0), *bak1-4* (Col-0), and *rbohD* (Col-0) mutants have been previously described [18,22,25].

### Chemicals

Synthetic flg22 peptide [21] was made by GenScript (Scotch Plains, NJ) and used at indicated concentrations. Horseradish Peroxidase (HRP; Sigma, catalog # P6782) was prepared as a 500x HRP stock solution by dissolving 10 mg/mL in sterile H_2_O. Aliquots (10-30 μL) of the 500x HRP stock solution were stored at -20˚C and used at a final concentration of 20 μg/mL. For the 500x Luminol stock solution, 17 mg Luminol (≥ 97% purity-HPLC; Sigma; catalog # A8511) was completely dissolved in 1 ml of 200 mM KOH and used at a final concentration of 0.2 μM. For example, 10 μl of each of the 500x stocks of luminol and HRP were added to 5 ml of resuspended bacterial solution. Because Luminol is light-sensitive, all solutions containing Luminol must be protected from light by wrapping tubes in aluminum foil. The 500x Luminol stock solution is made fresh prior to use and discarded daily.

### Bacterial preparation

Two days prior to ROS experiments, *Pseudomonas syringae* pv. *tomato* strain DC3000 (*Pto* DC3000) or *Pto hrcC*^*-*^ was streaked from glycerol stocks (stored at -80°C) onto King’s B medium (KBM) agar plates containing 50 μg ml^-1^ kanamycin and 30 μg ml^-1^rifampicin (*Pto* DC3000) or 30 μg ml^-1^rifampicin (*Pto hrcC*^*-*^) [38]. KMB plates containing bacteria were incubated for 36 to 48 hours at room temperature. Prior to elicitation, bacteria were scraped from plates and washed twice in sterile dH_2_O by repeated centrifugation at 10,000×*g* for 5 minutes. After the second wash step, a 1:10 dilution of the bacterial solution was made and its optical density (OD_600_) was measured using a spectrophotometer as a means to provide an approximate quantification of bacterial cell density [38,39]. The final bacterial elicitation solution was adjusted to an OD_600_ between 0.001 and 0.1, which under our conditions equated to 1 × 10^6^ to 10^8^ colony forming units (cfu)/mL based on serial dilution plating. The OD_600_ of the final bacterial elicitation solution was measured again to ensure accuracy of the dilution. To accurately compare the dose-dependency of the ROS response [Figure 3], bacteria were serially diluted from a stock solution.

### Measurement of apoplastic ROS production

One day before the ROS assay, leaf disks (1.1 cm^2^) from 4-5 week old plants were cut into two equal halves with a sharp razor blade to increase the cellular surface area exposed to elicitation solution, an important step for obtaining reproducible responses with less variability within and between experiment. Each leaf disc half was floated adaxial side up in an individual well of a 96-well microtiter plate (Costar; Fisher Scientific, catalog # 3912) containing 150 μl dH_2_O and then incubated overnight at 22°C in continuous light for 20 to 24 hours to reduce the wounding response. Prior to elicitation, the Elicitation Solution was prepared containing bacteria, Luminol and HRP. For a 10 ml Elicitation Solution, 20 μl of 500x HRP stock solution and 20 μl of the 500x Luminol stock solution is added to 10 ml of bacterial cells that have been already diluted to the desired concentration. For flg22-induced ROS production, flg22 peptide was used instead of bacteria in the Elicitation Solution. All Elicitation Solutions were kept at room temperature. Immediately prior to the elicitation, the incubating dH_2_O solution was carefully removed from each well avoiding any tissue damage or desiccation. Then using a multichannel pipetman, 100 μl of the Elicitation Solution was quickly added to each well containing leaf disc half. For Luminol-based ROS production, the plate was placed without delay into a GloMax^®^ 96-well microplate luminometer (Promega, Madison, USA) to measure *Pto*-induced ROS production between 0 and 80 minutes.

### Statistical analysis

Each experiment was done at least 3 independent times with similar results. Statistical significances based on unpaired two sample *t*-test were determined with Graph Pad Prism4 software (La Jolla, CA).

## Competing interests

The authors declare that they do not have any financial or other competing interests.

## Authors’ contributions

JMS performed experiments and made figures. JMS and AH designed experiments, analyzed and interpreted data, and wrote the manuscript. Both authors read and approved the final manuscript.

## Supplementary Material

Additional file 1 **Dose-dependent ROS production in response to synthetic flg22 peptide.** A: Time-course of ROS production in response to 1, 10 and 100 nM flg22 in wild-type Col-0 leaf disc halves (n = 16/treatment). B: Bar graph representation of peak ROS production for each flg22 concentration from experiment shown in A. To allow direct comparisons, all ROS experiments were performed in the same 96-well plate at the same time. Values are mean ± SE, means with different letters denote a significance difference (Two tailed student’s *t*-test, P < 0.0001). Experiment was repeated more than 3 times with similar results. RLU, Relative Light Units. Click here for file

Additional file 2 **Transient ROS production in response to live *Pto *** **DC3000, *Pto *** **DC3000 *avrRpm1 *** **and *Pto *** **DC3000 *avrRps4 *** **in Col-0.** A: Time-course of ROS production between *Pto* DC3000 and *Pto* DC3000 *avrRpm1* (n = 24/treatment). B: Timecourse of ROS production between *Pto* DC3000 and *Pto* DC3000 *avrRps4* (n = 24/treatment). C: ROS production induced by *Pto* DC3000 (black bar), *Pto* DC3000 *avrRpm1* (white bar) and *Pto* DC3000 *avrRps4* (gray bar) shown at peak ROS production (35 minutes post-elicitation) and as total Relative Light Units (RLUs) over 75 minutes following elicitation with indicated bacteria (n = 24/treatment). To allow direct comparisons, ROS experiments in (A, B and C) were performed in the same 96-well plate at the same time. Values are mean ± SE, means with different letters denote a significance difference while similar letters denote no significance (Two tailed student’s *t*-test, P ≥ 0.4084). For all experiments, bacterial solutions were used at OD600 = 0.1. Experiments were repeated more than 3 times with similar results. RLU, Relative Light Units. Click here for file

Additional file 3 **ROS production in response to live *Pto *** **DC3000 cells resuspended in dH**_**2**_**O or 10 mM MgCl**_**2**_**.** A: Time-course of ROS production in response to *Pto* DC3000 resuspended in sterile dH_2_O (closed symbols) or 10 mM MgCl_2_ (open sympols) in Col-0 leaf disc halves (n = 24/treatment). B: Comparison of peak ROS production between *Pto* DC3000 resuspended in sterile dH_2_O or 10 mM MgCl_2_ at the point of maximum ROS production (35 minutes post-elicitation from A). To allow for direct comparisons, all ROS experiments shown in (A and B) were performed in the same 96-well plate at the same time. Values are mean ± SE, means with different letters denote a significance difference while similar letters denote no significance (Two tailed student’s *t*-test, P = 0.59). For all experiments, bacterial solutions were used at OD600 = 0.1. Experiment was repeated more than 3 times with similar results. RLU, Relative Light Units. Click here for file

Additional file 4 **Lack of ROS production in *fls2 *** **mutant of different *Arabidopsis *** **ecotypes in response to live *Pto *** **DC3000 and *Pto hrcC***^**- **^**cells.** A and B: Time-course of ROS production in response to *Pto* DC3000 (A; closed symbols) or *Pto hrcC*^-^ (B; open sympols) in leaf disc halves of Col-0 (square), *fls2* (Col-0) null mutant (triangle) and Ws-0 (upside down triangle) (n = 16/treatment). C: Comparison of peak ROS production between *Pto* DC3000 or *Pto hrcC*^-^ in Col-0, *fls2* (Col-0) and Ws-0 shown at the point of maximum ROS production (35 minutes post-elicitation from A and B). To allow for direct comparisons, all ROS experiments shown in (A, B and C) were performed in the same 96-well plate at the same time. Values are mean ± SE, means with different letters denote a significance difference while similar letters denote no significance (Two tailed student’s *t*-test, P < 0.0001). For all experiments, bacterial solutions were used at OD600 = 0.1. Experiments were repeated more than 3 times with similar results. RLU, Relative Light Units. Click here for file
