# Supplementary material for: Rapid bioassay to measure early reactive oxygen species production in Arabidopsis leave tissue in response to living Pseudomonas syringae
Source: Plant Methods. 2014 Feb 26;10:6. doi: 10.1186/1746-4811-10-6 (PMC3941562; doi:10.1186/1746-4811-10-6)
Supplement: Additional file 1 — Dose-dependent ROS production in response to synthetic flg22 peptide. A: Time-course of ROS production in response to 1, 10 and 100 nM flg22 in wild-type Col-0 leaf disc halves (n = 16/treatment). B: Bar graph representation of peak ROS production for each flg22 concentration from experiment shown in A. To allow direct comparisons, all ROS experiments were performed in the same 96-well plate at the same time. Values are mean ± SE, means with different letters denote a significance difference (Two tailed student’s t-test, P < 0.0001). Experiment was repeated more than 3 times with similar results. RLU, Relative Light Units. [file 1746-4811-10-6-S1.pdf]

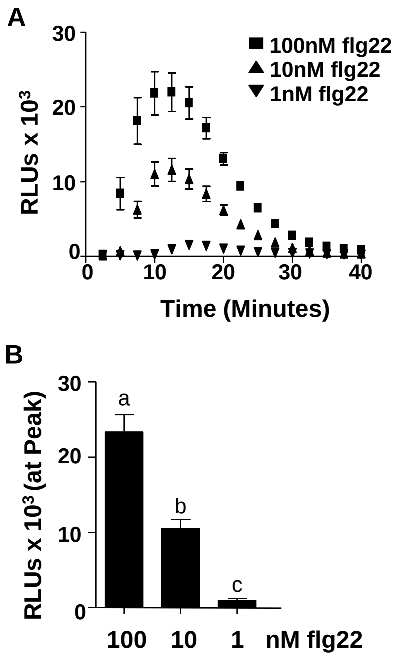

Additional file 1. Dose-dependent ROS production in response to synthetic flg22 peptide. A: Time-course of ROS production in response to 1, 10 and 100 nM flg22 in wild-type Col-0 leaf disc halves (n= 16/treatment). **B:** Bar graph representation of peak ROS production for each flg22 concentration from experiment shown in **A**. To allow direct comparisons, all ROS experiments were performed in the same 96-well plate at the same time. Values are mean ± SE, means with different letters denote a significance difference (Two tailed student's t-test, $P<0.0001$). Experiment was repeated more than 3 times with similar results. RLU, Relative Light Units.
