# Supplementary material for: Rapid bioassay to measure early reactive oxygen species production in Arabidopsis leave tissue in response to living Pseudomonas syringae
Source: Plant Methods. 2014 Feb 26;10:6. doi: 10.1186/1746-4811-10-6 (PMC3941562; doi:10.1186/1746-4811-10-6)
Supplement: Additional file 2 — Transient ROS production in response to live Pto DC3000, Pto DC3000 avrRpm1 and Pto DC3000 avrRps4 in Col-0. A: Time-course of ROS production between Pto DC3000 and Pto DC3000 avrRpm1 (n = 24/treatment). B: Timecourse of ROS production between Pto DC3000 and Pto DC3000 avrRps4 (n = 24/treatment). C: ROS production induced by Pto DC3000 (black bar), Pto DC3000 avrRpm1 (white bar) and Pto DC3000 avrRps4 (gray bar) shown at peak ROS production (35 minutes post-elicitation) and as total Relative Light Units (RLUs) over 75 minutes following elicitation with indicated bacteria (n = 24/treatment). To allow direct comparisons, ROS experiments in (A, B and C) were performed in the same 96-well plate at the same time. Values are mean ± SE, means with different letters denote a significance difference while similar letters denote no significance (Two tailed student’s t-test, P ≥ 0.4084). For all experiments, bacterial solutions were used at OD600 = 0.1. Experiments were repeated more than 3 times with similar results. RLU, Relative Light Units. [file 1746-4811-10-6-S2.pdf]

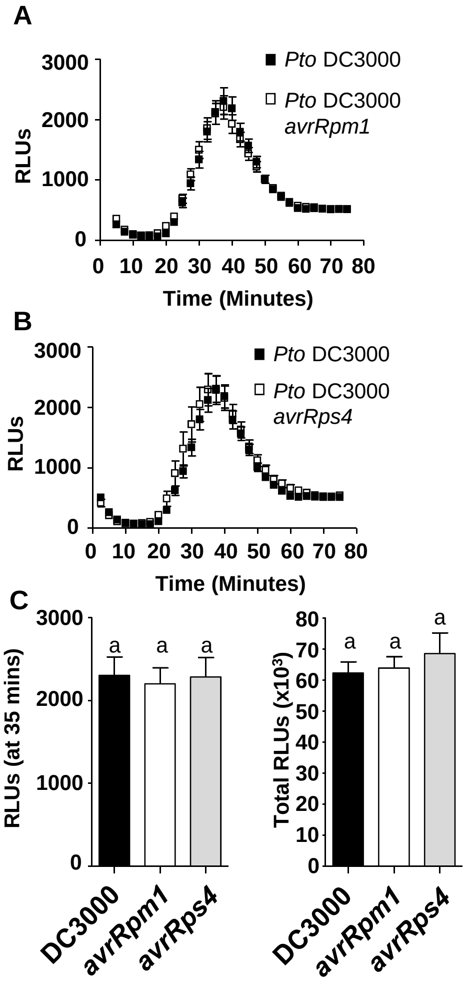

Additional file 2. Transient ROS production in response to live *Pto* DC3000, *Pto* DC3000(*avrRpm1)* and *Pto* DC3000(*avrRps4)* in Col-0. A: Time-course of ROS production between *Pto* DC3000 and *Pto* DC3000 *avrRpm1* (n=24/treatment). **B:** Time-course of ROS production between *Pto* DC3000 and *Pto* DC3000 *avrRps4* (n=24/treatment). **C:** ROS production induced by *Pto* DC3000 (black bar), *Pto* DC3000 *avrRpm1* (white bar) and *Pto* DC3000 *avrRps4* (gray bar) shown at peak ROS production (35 minutes post-elicitation) and as total Relative Light Units (RLUs) over 75 minutes following elicitation with indicated bacteria (n=24/treatment). To allow direct comparisons, ROS experiments in (**A**, **B** and **C**) were performed in the same 96-well plate at the same time. Values are mean ± SE, means with different letters denote a significance difference while similar letters denote no significance (Two tailed student's t-test, P≥0.4084). For all experiments, bacterial solutions were used at OD_{600}=0.1. Experiments were repeated more than 3 times with similar results. RLU, Relative Light Units.
