# Supplementary material for: Rapid bioassay to measure early reactive oxygen species production in Arabidopsis leave tissue in response to living Pseudomonas syringae
Source: Plant Methods. 2014 Feb 26;10:6. doi: 10.1186/1746-4811-10-6 (PMC3941562; doi:10.1186/1746-4811-10-6)
Supplement: Additional file 3 — ROS production in response to live Pto DC3000 cells resuspended in dH 2 O or 10 mM MgCl 2 . A: Time-course of ROS production in response to Pto DC3000 resuspended in sterile dH2O (closed symbols) or 10 mM MgCl2 (open sympols) in Col-0 leaf disc halves (n = 24/treatment). B: Comparison of peak ROS production between Pto DC3000 resuspended in sterile dH2O or 10 mM MgCl2 at the point of maximum ROS production (35 minutes post-elicitation from A). To allow for direct comparisons, all ROS experiments shown in (A and B) were performed in the same 96-well plate at the same time. Values are mean ± SE, means with different letters denote a significance difference while similar letters denote no significance (Two tailed student’s t-test, P = 0.59). For all experiments, bacterial solutions were used at OD600 = 0.1. Experiment was repeated more than 3 times with similar results. RLU, Relative Light Units. [file 1746-4811-10-6-S3.pdf]

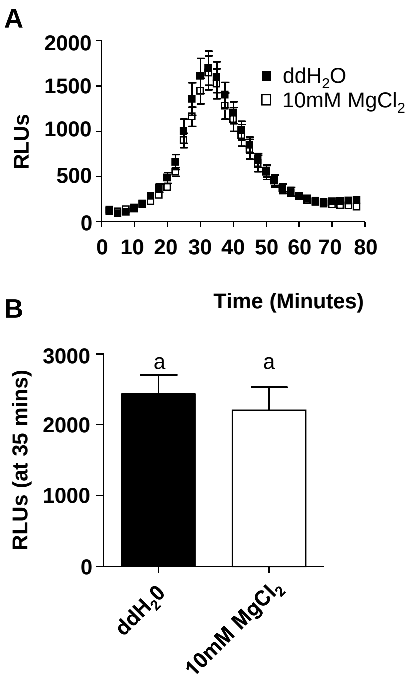

Additional file 3. ROS production in response to live *Pto* DC3000 cells re-suspended in ddH$_2$O or 10mM MgCl$_2$. A: Time-course of ROS production in response to *Pto* DC3000 resuspended in sterile ddH20 (closed symbols) or 10mM MgCl2 (open sympols) in Col-0 leaf disc halves (n=24/treatment). **B**: Comparison of peak ROS production between *Pto* DC3000 resuspended in sterile ddH$_2$0 or 10mM MgCl$_2$ at the point of maximum ROS production (35 minutes post-elicitation from **A**). To allow for direct comparisons, all ROS experiments shown in (**A** and **B**) were performed in the same 96-well plate at the same time. Values are mean ± SE, means with different letters denote a significance difference while similar letters denote no significance (Two tailed student's t-test, P=0.59). For all experiments, bacterial solutions were used at OD$_{600}$=0.1. Experiment was repeated more than 3 times with similar results. RLU, Relative Light Units.
