# Supplementary material for: Rapid bioassay to measure early reactive oxygen species production in Arabidopsis leave tissue in response to living Pseudomonas syringae
Source: Plant Methods. 2014 Feb 26;10:6. doi: 10.1186/1746-4811-10-6 (PMC3941562; doi:10.1186/1746-4811-10-6)
Supplement: Additional file 4 — Lack of ROS production in fls2 mutant of different Arabidopsis ecotypes in response to live Pto DC3000 and Pto hrcC - cells. A and B: Time-course of ROS production in response to Pto DC3000 (A; closed symbols) or Pto hrcC- (B; open sympols) in leaf disc halves of Col-0 (square), fls2 (Col-0) null mutant (triangle) and Ws-0 (upside down triangle) (n = 16/treatment). C: Comparison of peak ROS production between Pto DC3000 or Pto hrcC- in Col-0, fls2 (Col-0) and Ws-0 shown at the point of maximum ROS production (35 minutes post-elicitation from A and B). To allow for direct comparisons, all ROS experiments shown in (A, B and C) were performed in the same 96-well plate at the same time. Values are mean ± SE, means with different letters denote a significance difference while similar letters denote no significance (Two tailed student’s t-test, P < 0.0001). For all experiments, bacterial solutions were used at OD600 = 0.1. Experiments were repeated more than 3 times with similar results. RLU, Relative Light Units. [file 1746-4811-10-6-S4.pdf]

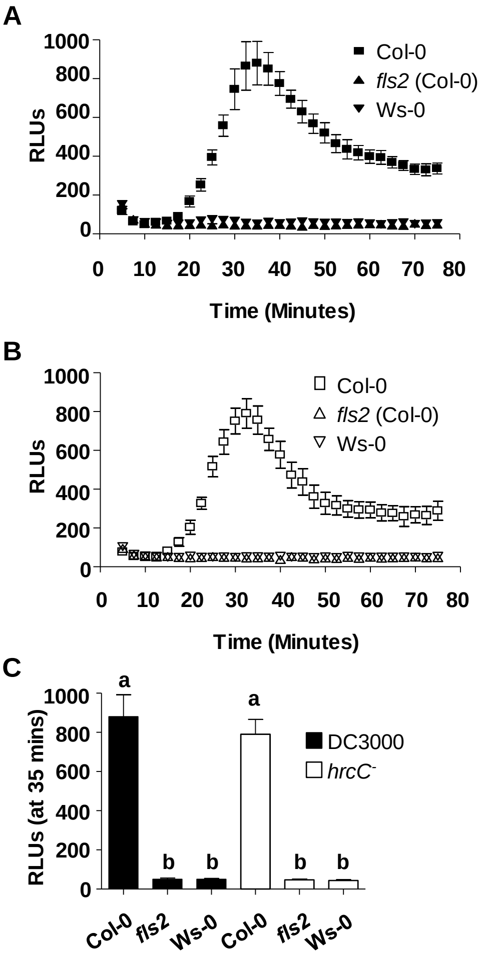


**Additional file 4. Lack of ROS production in *fls2* mutant of different *Arabidopsis* ecotypes in response to live *Pto* DC3000 and *Pto hrcC*$^{-}$ cells. A and B:** Time-course of ROS production in response to *Pto* DC3000 (**A**; closed symbols) or *Pto hrcC*$^{-}$ (**B**; open sympols) in leaf disc halves of Col-0 (square), *fls2* (Col-0) null mutant (triangle) and Ws-0 (upside down triangle) (n=16/treatment). **C**: Comparison of peak ROS production between *Pto* DC3000 or *Pto hrcC*$^{-}$ in Col-0, *fls2* (Col-0) and Ws-0 shown at the point of maximum ROS production (35 minutes post-elicitation from **A** and **B**). To allow for direct comparisons, all ROS experiments shown in (**A, B** and **C**) were performed in the same 96-well plate at the same time. Values are mean ± SE, means with different letters denote a significance difference while similar letters denote no significance (Two tailed student's t-test, P<0.0001). For all experiments, bacterial solutions were used at $OD_{600}$=0.1. Experiments were repeated more than 3 times with similar results. RLU, Relative Light Units.
